# Composition, physicochemical property and base periodicity for discriminating lncRNA and mRNA

**DOI:** 10.6026/973206300191145

**Published:** 2023-12-31

**Authors:** Prasad Rajesh, Annangarachari Krishnamachari

**Affiliations:** 1 School of Computational and Integrative Sciences, Jawaharlal Nehru University, New Delhi, 110067, India

**Keywords:** lncRNA, mRNA, Bioinformatics, physicochemical feature, machine learning, computational biology

## Abstract

Annotation of genome data with biological features is a challenging problem. One such problem deals with distinguishing lncRNA from mRNA. In this study, three groups of classification features, namely base periodicity, physicochemical property and nucleotide compositions were considered. We are attempting to propose a simple neural network model to obtain better results using judicious combination of the above said sequence features. Our approach uses balanced dataset, simple prediction model and use of limited features in distinguishing lncRNA and mRNA. Accordingly (a) two properties of base periodicity: peak power spectrum of the signal and noise-to-signal ratio (SNR) of this peak signal (b) three physicochemical properties: solvation, stacking and hydrogen-bonding energy and (c) all dinucleotides and trinucleotides compositions were used. Classification was performed by considering features independently followed by combining these properties for improvement. Classification metric was used to compare the result for seven eukaryotic organisms for various combinations of features. Nucleotide compositions combined with physicochemical property or base periodicity group of features becomes a strong classifier with more than 99 percentage accuracy. Base periodicity analysis with SNR can be used as discriminating feature of lncRNA from mRNA.

## Background:

Gene transcription is one of the very important processes, where information embedded in DNA is transcribed into different types of RNA. Only one type of RNA is responsible for coding proteins, and the rest are non-coding but are involved in other cellular activities and at various stages of gene regulation [1]. Specific orientation and spatial stability of DNA is very important for storage and retrieval of correct information [2]. Further, under noncoding several categories were characterized like long noncoding RNA (lncRNAs), microRNAs (miRNA), small interfering RNAs (siRNAs), ribosomal RNAs (rRNAs), etc. There is a growing interest in understanding the functionality of lncRNA. Since lncRNA acts as a biomarker of various diseases, studies related to lncRNA are undertaken in a big way [3].

There are a small number of experimentally annotated sequences known so far in the publicly available databases which create an uphill task in developing a good software prediction tool. In this field LncFinder software package, introduced in 2019 by Han,S. *et al.,* is employed for predicting lncRNA [4]. Our predictive model not only distinguishes between lncRNA and mRNA but also contributes meaningfully, highlighting the importance of our work in complementing the capabilities of LncFinder. Heterogeneity in lncRNA is another problem that creates obstacles in proposing standard methods for characterization [5]. Researchers employ an integrative approach i.e. combining experimental and *in silico* approach to identify lncRNA.,

Spatial arrangement of DNA sequence depends on many physicochemical factors, like ionic interaction, hydrogen bonding, base stacking, pH, salt concentration, superposing, etc. [6]. Although all physicochemical properties are important, hydrogen bonding, stacking, and solvation based energies play critical roles in maintaining the structural integrity of DNA molecules [7]. Despite biochemical similarities, lncRNA and mRNA have many distinguishable factors, like distinct sequence patterns. Since mRNA sequences have open reading frames and CDS arrangement, their pattern differs from non-specific lncRNA arrangements [1].

In this study, we have considered three parameters (1) di-base and tri-base nucleotide compositions (2) the physicochemical property (solvation energy, stacking energy, and hydrogen bonding energy) of di and tri-base and (3) base periodicity within DNA sequences. Analysis of sequences from different organisms verifies this fact in bacteria that the 3-base periodicity is embedded in protein-coding sequences. This feature is weak in non-coding sequences [8]. This can be seen in the frequency domain through the Fourier transform [9].

The Artificial neural network model (ANN) is a popular framework in sequence analysis tasks because it captures symbolic patterns more intelligently than conventional statistical methods [10]. In the current study, we have built a sequence-based binary classification model for eukaryotic lncRNA and mRNA, both for standalone features and in combination with three feature classes. Many attempts were made using computational method prediction or classification of lncRNA and mRNA, reader may also refer about the comparative performance of various prediction tools [11].

In this study, we have considered seven model organisms for lncRNA and mRNA sequences. All these species have differences in the number of lncRNA and experimental mRNA data. Several features were derived from the basic properties i.e., composition, Physicochemical energy and periodicity, in this way, we have a total 88 features for this model. Naturally dimensionality reduction needed attention. Considering available data pertaining to lncRNA and mRNA, our study tried to utilize the embedded properties to make a distinction and a step to improve the classification performance. Our study is novel in preparing a good prediction model with a simple neural network setup with a combination of features.

## Materials and Methods:

## Data source:

The sequences of lncRNA for all seven organisms have been taken from the Non-Code database of version V5.0. [12]. Experimental sequences of mRNA were filtered from the transcript files publicly available at NCBI. Also, these sequences of lncRNA and mRNA were randomly chosen from all organisms to construct a combined (mix) dataset. Experimental values of Dinucleotides physicochemical energy are listed in Supplementary Table S1 and Experimental values of trinucleotides physicochemical energy in Supplementary Table 2 of additional file 1. The number of lncRNA and mRNA sequences of the chosen eukaryotes for our study is listed in Table 1.

Composition based features:(a) PCA-based dinucleotide compositions, (b) PCA-based trinucleotide compositions, (2) Physicochemical energy-based features: (a) solvation energy, (b) stacking energy, (c) hydrogen bonding energy of dinucleotide and trinucleotide sequences and (3) Base periodicity-based features: (a) peak power spectrum (b) SNR. Following section covers these features in detail.

## Physicochemical properties of dinucleotides and trinucleotides:

Experimental values of physicochemical energy have been derived from a series of MD simulations for all possible tetra-nucleotide sequences [13]. Further, solvation energy, stacking energy, and hydrogen bonding energy for all possible trinucleotide combinations have been derived from their average occurrence in tetra-nucleotides. These properties for trinucleotides have been further mapped to dinucleotides [13,14]. Sequence can be modelled as a separate Markov chain and first order transition matrix can be constructed [15].

The transition matrix of trinucleotides or dinucleotides is, P^k^ = p(s_i_/s_j_), where p is the probability of transition from the state s_i_ to state s_j_ of k_th_ sequence of lncRNA/mRNA, s_j_ belongs to 4096 states space for trinucleotide and 256 states of dinucleotides. The procedure for construction is as follows [14-16]. Detailed description of the method for calculation of physicochemical properties features of dinucleotides and trinucleotides are added in Supplementary figure S1 in additional file 1.

## Base periodicity of lncRNA and mRNA:

In this study, we have analyzed the peak of the power spectrum and SNR of peak spectrum. In bioinformatics, base periodicity has been explored to unravel biological features. Fourier spectrum of lncRNA and mRNA is defined using binary indicator function as described in Method 1 of additional file 2 [8].

## Principal component analysis (PCA) of Dinucleotides and trinucleotides:

Counting the number of all 2-base, and 3-base compositions for all sequences of lncRNA and mRNA separately, this creates 64-dimensional data for trinucleotides and 16-dimensional for dinucleotides. To overcome the computational complexity, PCA is used on this to obtain 15 principal components (pc) for each category i.e. dinucleotide composition and trinucleotide compositions. In this way, the total dimension is reduced to 30 (15 pc each for trinucleotides and dinucleotides compositions).

## Data preparation:

Since, we have unequal amount of data for the classification task. Hence, two approaches are undertaken: (1) randomly select data from the major class and matched with same number as minor class, here major refers to the one having higher training examples in the chosen species. (2) Using SMOTE to build the model, SMOTE systematically over samples data in the minor class. SMOTE algorithm draws a line segment between neighbouring minority instances that lie in the minority class. SMOTE selects randomly these line segments and generates new minority instances [17].

## Neural network model:

The number of neurons in the input layer and hidden layers varies according to the dimensionality of input data for the network. There are five types of single features DP, TP DC, TC, and BP. All other features are a combination of these five individual features. For example, TPTC is a combination of features TP (mean weighted Trinucleotide physicochemical energy) and TC (Principle component of trinucleotides). We select 15-dimensional data in 16 different proportions for all 16 types of feature combinations from the total data sets as mentioned in Table 2.

For our study, we have three major feature groups. We have constructed sixteen neural network model setups as shown in Supplementary Table S3 with selectively chosen features. Details of the data preparation flow chart were explained in supplementary Figure S2 and supplementary Table S3. The architecture of all 16 neural networks is listed in Supplementary Table S3.

## Results:

Genome sequence data were generated in a faster manner, which results in huge amounts of data to be analyzed. Only a computational approach is the solution for this problem and to give biological meaning to raw data. As described in method section we consider seven organisms and their sequences belonging to lncRNA and mRNA. The analysis proposed on the sequences based on the above said properties are described and how to discriminate them using a simple prediction model. In our analysis dealing with lncRNA and mRNA two types of approaches followed in removing data imbalance. For the first case, random data were selected from the one having a higher sample to equal the number of sequences having fewer ones. In the second case, data imbalance is achieved using the SMOTE algorithm as described in the material and method section.

## Periodicity spectrum:

**Figure 1:**
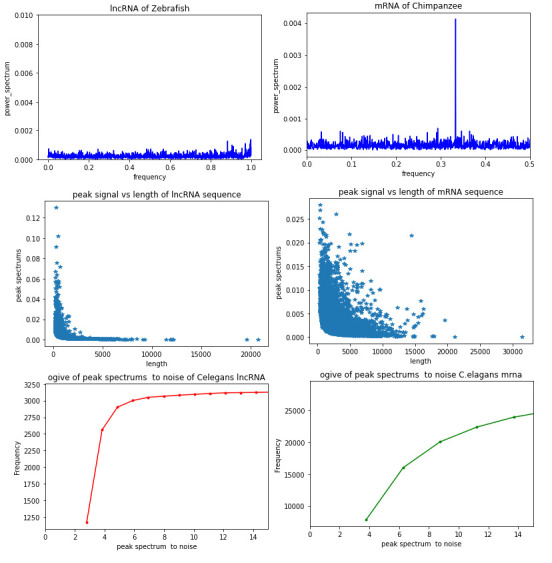
Variation of the peak power spectrum and SNR for lncRNA and mRNA. (a) Power spectrum of mRNA for Chimpanzee mRNA (NM 001008975.1) and (b) for Power spectrum of zebrafish lncRNA (NONDRET013003; (c) and (d) are Scatter plots of Chicken mRNA and lncRNA respectively varies with length. (e and 1.f are given plots of SNR for C. elegens mRNA and lncRNA respectively.

The highest value of the periodicity spectrum is considered as peak w.r.t certain frequency, Figure 1a and b depicts the spectrum for mRNA and lncRNA and easy to see that a prominent peak is absent in lncRNA. Value of Peaks of lncRNA and mRNA sequences are shown in Figure 1c and d as a scatter plot w.r.to its length. It can be observed that the peak spectrum at 1/3 decreases with increased length, in both mRNA and lncRNA. However, the average peak in mRNA is higher than that of lncRNA as expected. Ogive plot given in Figure 1e and f, it is observed that SNR is higher in mRNA as compared to lncRNA. Majority of the SNR for mRNA is above 4 whereas SNR for lncRNA is below 4. This observation may be useful for discrimination purposes.

**Figure 2:**
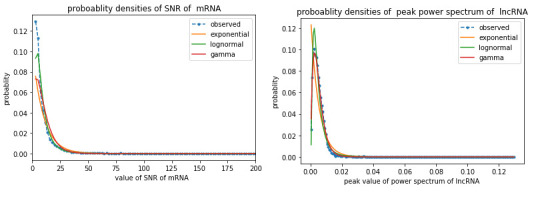
Fitting of candidate distribution with MLE parameter; (a) Distribution of SNR for Chicken mNRA and (b) for Chicken lncRNA.

One can suggest positively skewed distributions for maximum peak spectrum as well as SNR for all considered organisms. Lognormal, gamma and exponential distributions are generally used to model many processes in molecular biology [18]. In this study, we have fitted lognormal, gamma, and exponential distributions for SNR and peak spectrum. It can be seen from Figure 2 that lognormal distribution shows best fit. Details of lognormal fit described in Supplementary Figure S3 of additional file 2, and MLE values are listed in Supplementary Table S4. In the feature combination process, we have selected 4, 5, 6, 7, 8, 12 and 15 PCs from the transformed PC space and more than 95 percent variability is retained in the PCs. These are listed in the Supplementary Table S5.

In this study, we have used the binary cross-entropy function.

Loss(y, y^) = -y log(y^) - (1 - y). log(1 - y^)

**Figure 3:**
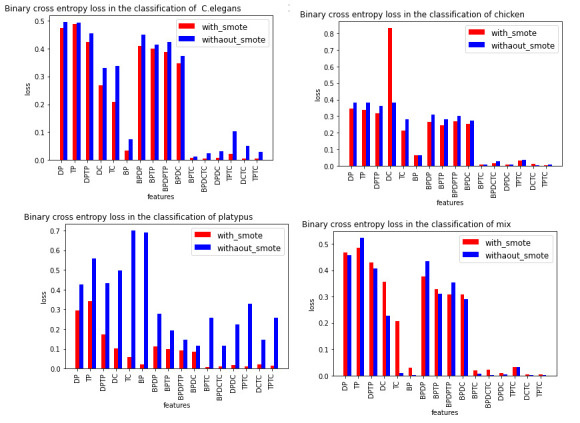
Binary cross entropy loss with and without SMOTE (a) *C. elegans*, (b) Chicken, and (c) for mix of lncRNA/mRNA of *C. elegans*, chicken chipmanzee and platypus.

Where y is true label [0 and 1], and y^ is predicted label usually between 0 and 1 Figure 3a, b and c shows binary cross entropy loss for three of 7 considered organisms namely chicken, chimpanzee, and platypus respectively, and a mix of their sequences shown in Figure 3d. There are three regimes in plots. The left regime is the loss of model for individual features like DP, TP, BP, etc. Middle regime in the plot for combined, physicochemical properties and base periodicity of dinucleotides as well as trinucleotides is noted. The right regime for a combination of trinucleotide and dinucleotide composition with base periodicity and physicochemical properties is also noted. In the case of *C. elegans*, the number of experimental mRNA sequences is larger than the number of lncRNA sequences. However, in the case of chickens, there are more lncRNA sequences than experimental mRNA sequences. There are fewer experimental mRNA sequences available in the database for Platypus as compared to lncRNA, this leads to the model having more bias and therefore generalization not good with fewer data points.it is evident from Figure 3d.

Classification Metrics of all 16 feature combinations for 6 considered organisms using approach 2 (with SMOTE) listed in supplementary Table S6, Table S7, Table S8 in the additional file 3. (metrics for Chimpanzee and *C. elegans* in supplementary Table S6, Cow and Platypus in supplementary Table S7, and Zebrafish and Arabidopsis thaliana in supplementary Table S8).It seems that the model performances are better with nucleotide composition than the physicochemical property (dinucleotide and trinucleotide).

However, performance got enhanced significantly when combining dinucleotide composition with trinucleotide composition features. Overall it can be said that any single or engineered features combined with trinucleotide composition provide remarkable improvement. Some combined features like DCTC, BPDC, BPTC, BPDCTC, DPDC, TPTC, and DPTPDCTC have a remark-able performance of up to 99 percent accuracy. The classification metric scores of the mixtures of these species are approximately similar to the scores for individual species as shown in bar chart of supplementary Figure S4 (Supplementary Material).

Classification metrics for the organisms *C. elegans*, chimpanzees, platypus, and for the mixed sequences of these three organisms, have been compiled using both approaches with and without SMOTE. Metrics of *C. elegans* and platypus are listed in Table 3. Classification metrics of chicken and mix sequences of these three species are listed in Supplementary Table S9. In these tables, on the left of '/' is the classification metric for the balanced datasets (using SMOTE), while on the right are the metric values for the balanced datasets (without using SMOTE). It can be seen that there is a small difference in metrics between the two approaches in performance for individual features, whereas the metric values difference between the two approaches is relatively less in the case of combined features.

## Discussion:

The objective of genome annotation is to characterize the DNA sequence makeup in terms of biological features. It is a difficult task because DNA sequences have embedded information at different lengths, scales, heterogeneity, order, compositions, etc. Codons, which are a trinucleotide combination, can distinguish coding RNA from other types. Similarly, many sequence features can be utilized to identify other RNAs. Several approaches and algorithms were proposed to classify lncRNA and mRNA [11]. For the data imbalance case SMOTE may do over fitting to some extent but we do not have other choices. This study is exhaustive in nature and introduces a machine learning-based binary classification approach that integrates three distinct sets of properties for discriminating between lncRNA and mRNA sequences. When we consider these three properties i.e. compositions, physicochemical property and base periodicity individually for the clarification purpose, they showed poor results. Among three trinucleotide compositions is the best choice. However, when model with combination of composition, periodicity and physicochemical property, it showed good benchmark performance results.

## Conclusion:

Base periodicity is better classifier than the model based on physiochemical properties. However, the model based on composition is better than the base periodicity. Model with combined features perform better than that of those individual's case. From the analysis we conclude that the trinucleotide composition is best individual classifier. However, it enhances performance when combined with dinucleotide compositions base periodicity and physicochemical properties. This model attains high accuracy of about 99 percent and provides valuable insights into the structural aspect of gene sequences.

## Limitation:

The lncRNA data were downloaded from public database which contains experimentally validated and computationally predicted lncRNA. Hence, one must keep this fact while studying the metrics.

## Author contributions:

A.K- Conceptualization, methodology, formal analysis, writing-review and editing, supervision. RP- Conceptualization, formal analysis, investigation, data curation, writing-original draft. All authors read and approved the final manuscript.

## Funding:

Not applicable

## Availability of data and materials:

lncRNA sequence - NONCODE database (http://v5.noncode.org/download.php) mRNA sequence - Experimental sequences of mRNA were filtered from the transcript files publicly available at NCBI (https://www.ncbi.nlm.nih.gov/genome/), then filter experimental mRNA only.

## Ethics approval and consent to participate:

The Authors declare that no approval is required for the current study.

## Competing interests:

The Authors declare that they have no competing interests.

## Figures and Tables

**Table 1:** lncRNA and mRNA sequences of eukaryotic organisms

**Model organisms**	**lncRNA**	**mRNA**
Chimpanzee	18002	2025
Platypus	11208	264
Zebrafish	4850	15392
*C. elegans*	3152	28532
Chicken	12848	8377
Cow	23513	13307
*Arabidopsis thaliana*	3762	48145
Combined set	77335	116042

**Table 2:** Feature combination and feature selection

**S. No.**	**Types of features and their combination **	**No. of feature selected**
1	Dinucleotides Physicochemical energy (DP)	3 (3 features from DP)
2	Trinucleotide physicochemical Energy (TP)	3 (3 features from DP)
3	Dinucleotide and Trinucleotide Physicochemical energy	3+3=6 (3 features from DP and three features from TP)
4	PCA-based Dinucleotide composition (DC)	15 (15 features from DC)
5	PCA-based Trinucleotide composition (TC)	15 (15 features from TC)
6	Base periodicity (BP)	2 (2 features from BP)
7	Base periodicity and Dinucleotide Physicochemical energy (BPDP)	2+3= 5 (2 features from BP and 3 features for DP)
8	Base periodicity and Trinucleotide Physicochemical energy (BPTP)	2+3= 5 (2 features from BP and 3 features from TP)
9	Base periodicity, Dinucleotide physicochemical energy and Trinucleotide (BPDPTP)	2+3+3= 8 (2 features from BP, 3 features from DP and Three features from
10	Base periodicity and PCA-based Dinucleotide Composition (BPDC)	2+13=15 ( 2 features from BP and 13 feature from DC)
11	Base periodicity and PCA based Trinucleotide compositions (BPTC)	2+13=15 (2 features from BP and 2 feature from TC)
12	Base periodicity, PCA based Dinucleotide composition and PCA based Trinucleotides composition (BPDCTC).	2+7+6=15 ( 2 features from BP ,7 features from DC and 6 feature from TC)
13	Dinucleotide Physicochemical energy and PCA based Dinucleotides composition (TPTC)	3+12 =15 (3 features from TP and 12 features from TC)
14	Trinucleotide physicochemical energy and PCA based Trinucleotide composition (DCTC)	3+12=15 (3 features from DC and 12 features from TC)
15	PCA based Dinucleotide compositions and PCA based Trinucleotide compositions (DCTC)	8+7 =15 (8 features from DC and 7 features from TC)
16	Trinucleotide physicochemical energy, PCA based Trinucleotide compositions, Dinucleotide physicochemical energy and Dinucleotide compositions. (TPTCDPDC)	3+4+3+5 =15 (3 feature from TP, 4 features from TC, 3 features from DP and 5 features from DC

**Table 3:** The classification metrics

**Organism**	***C. elegans***					
**Combination**	**Accuracy**	**Precision**	**Recall**	**Roc**	**Prc**	**F1 score**
DP	0.7788/0.7709	0.8004/0.8245	0.7444/0.7037	0.8595/0.8569	0.8448/0.8683	0.7714/0.759325
TP	0.7915/0.76148	0.7602/0.8449	0.8513/0.6558	0.8763/0.8528	0.8680/0.8409	0.8027/0.738435
DPTP	0.8020/0.7709	0.7996/0.8213	0.7955/0.6990	0.8885/0.8670	0.8887/0.8706	0.7958/0.75523
DC	0.8807/0.87638	0.8712/0.9028	0.8943/0.85970	0.9556/0.9344	0.9567/0.9418	0.8823/0.88072
TC	0.9176/0.876	0.9112/0.8629	0.9264/0.8881	0.9731/0.9408	0.9708/0.9194	0.9184/0.87531
DCTC	0.9888/0.9793	0.9887/0.9811	0.9892/0.9781	0.9988/0.9948	0.9968/0.99541	0.9889/0.97954
BP	0.8030/0.7812	0.7884/0.7968	0.8321/0.7799	0.8916/0.8791	0.8793/0.8663	0.8097/0.78825
BPDP	0.8106/0.79318	0.8214/0.7533	0.7881/0.8421	0.8992/0.8943	0.8959/0.8737	0.8044/0.79522
BPTP	0.8253/0.8090	0.82147/0.78930	0.8314/0.8143	0.9079/0.8931	0.9011/0.8879	0.8314/0.8016
BPDPTP	0.8345/0.8225	0.8247/0.8681	0.8507/0.7571	0.9245/0.91287	0.9267/0.9096	0.8375/0.8088
BPDC	0.9978/0.9976	0.9989/0.9955	0.9968/1.0	0.9997/0.9991	0.99970/0.9984	0.9978/0.997744
BPTC	0.9986/0.9968	0.9982/0.9969	0.9991/0.9969	0.9999/	0.99980/0.9968	0.9986/0.9969
BPDCTC	0.9993/0.9952	0.99982/0.9969	0.99894/0.9969	0.99964/0.9987	0.99978/0.9977	0.99938/0.9969
DPDC	0.9940/0.9952	0.9930/0.9716	0.9947/0.9824	0.9993/0.9935	0.9989/0.9882	0.9938/0.97697
TPTC	0.9991/0.9936	0.9982/0.9937	1.000/0.9937	0.9997/0.9974	0.9994/0.9962	0.9991/0.9937
DPTPDCTC	0.9999/0.9960	0.9999/0.9935	0.9993/0.9983	0.9997/0.9990	0.9999/0.9981	0.9995/0.99589
**Organism**	**Platypus**					
DP	0.87801/0.8113	0.89061/0.8333	0.8579/0.76923	0.9434/0.8557	0.94967/0.8951	0.8730/0.799984
TP	0.8612/0.8584	0.8950/0.8333	0.8193/0.8510	0.8949/0.9051	0.9284/0.9229	0.8552/0.842056
DPTP	0.9288/0.8679	0.9301/0.9200	0.9272/0.8214	0.9820/0.9185	0.9839/0.9362	0.9291/0.86768
DC	0.9591/0.7735	0.9704/0.7555	0.9704/0.7234	0.9922/0.80814	0.9934/0.84018	0.9591/0.739101
TC	0.9830/0.6415	0.9875/0.64814	0.9788/0.6481	0.99761/0.7079	0.99789/0.6648	0.9831/0.648119
DCTC	0.9946/0.58490	0.9964/0.5671	0.9929/0.7169	0.9984/0.63581	0.9987/0.6422	0.99447/0.63326
BP	0.96275/0.9245	0.93993/0.8906	0.98887/0.9827	0.9904/0.9565	0.98989/0.9027	0.963782/0.934385
BPDP	0.96632/0.97169	0.95116/0.9607	0.98302/0.9800	0.9912/0.99285	0.99015/0.9915	0.96683/0.97025
BPTP	0.9694/0.9716	0.96396/0.9499	0.97405/1.0	0.99481/0.9915	0.9949/0.9913	0.96898/0.9743
BPDPTP	0.97033/0.952830	0.96236/0.8999	0.97907/1.0	0.99315/0.9974	0.98955/0.9965	0.970646/0.94731
BPDC	0.99776/0.8867	0.99954/0.9259	0.99594/0.8620	0.999970.9601	0.99997/0.9649	0.96683/0.8928
BPTC	0.99866/0.9811	1/0.9830	0.99726/0.98305	0.9999/0.99026	0.9999977/0.9948	0.99933051/0.993747
BPDCTC	0.99955/0.9528	0.99954/0.9591	0.999547/0.9399	0.99976/0.9896	0.999852/0.98978	0.9995478/0.94940
DPDC	0.9977/0.89622	0.9990/0.910714	0.9963/0.8947	0.9992/0.93340	0.9995/0.9098	0.9977/0.902629
TPTC	0.9968/0.9528	0.9942/0.9473	0.99955/0.96428	0.9971/0.9853	0.9941/0.9828	0.9968/0.9281
DPTPTC	0.9975/0.9339	0.9981/0.95081	0.9967/0.9354	0.9995/0.96151	0.9996/0.3587	0.9974/0.94304

## References

[1] Luisa Stateollo (2021). Nature Reviews Molecular Cell Biology..

[2] Ceze L (2019). Nature Reviews Genetics.

[3] Fattahi S (2020). Journal of cellular physiology..

[4] Siyu H (2019). Briefings in Bioinformatics..

[5] Zhao Z (2021). Oncogene..

[6] Sharma D (2020). IET nanobiotechnology..

[7] Sundaralingam M, Ponnuswamy PK (2004). Biochemistry..

[8] Tiwari S (1997). Bioinformatics..

[9] Voss RF (1992). Physical review letters..

[10] Chakraborty I (2017). Journal of Information Technology & Software Engineering.

[11] Ammunt T (2022). Briefings in Functional Genomics..

[12] Fang SS (2018). Nucleic acids Research..

[13] Pasi M (2014). Nucleic Acids Research..

[14] Jaiswal AK (2019). Computational Biology and Chemistry..

[15] Wang J, Sridhar H (2005). BMC Bioinformatics..

[16] https://icc2023.ieee-icc.org/.

[17] Fernandez A (2018). J. Artif. Intell. Res..

[18] Thangjai W, Niwitpong SA (2020). Iran J Sci Technol Trans Sci.

