# Supplementary material for: Composition, physicochemical property and base periodicity for discriminating lncRNA and mRNA
Source: Bioinformation. 2023 Dec 31;19(12):1145–52. doi: 10.6026/973206300191145 (PMC10794758; doi:10.6026/973206300191145)
Supplement: Data 1 [file 973206300191145S1.pdf]

Supplementary Materials

Supplementary Table 1: Experimental values of Dinucleotides physicochemical energies.

Dinucleotides	Hydrogen bonding energy (kcal/mol/bp)	Stacking energy (kcal/mol/bp)	Solvation energy (kcal/mol/bp)
AA	-5.44	-26.71	-171.84
AC	-7.14	-27.73	-171.11
AG	-6.27	-26.89	-174.93
AT	-5.53	-27.20	-173.70
CA	-7.01	-27.15	-179.01
CC	-8.48	-26.28	166.76
CG	-8.05	-27.93	-176.88
CT	-6.27	-26.89	-174.93
GA	-7.80	-26.78	-167.60
GC	-8.72	-28.13	-165.58
GG	-8.48	-26.28	-166.76
GT	-7.14	-27.73	-171.11
TA	-5.83	-26.90	-174.35
TC	-7.80	-26.78	-167.60
TG	-7.01	-27.15	-179.01
TT	-5.44	-26.71	-171.84

Supplementary Table 2: Experimental values of Trinucleotides physicochemical energies.

Trinucleotides	Hydrogen bonding energy (kcal/mol/bp)	Stacking energy Energy (kcal/mol/bp)	Solvation energy (kcal/mol/bp)
CCC	-10.7000	-56.1505	-329.400
CCG	-11.2025	-54.7810	-349.200
CCT	-13.9495	-56.2000	-344.700
CCA	-13.9830	-55.8865	-348.000
CGC	-10.7670	-52.9000	-345.600
CGG	-11.2025	-54.7810	-349.200
CGT	-13.0450	-53.3785	-349.700
CGA	-12.6765	-54.3850	-351.300
CTC	-14.2845	-55.7875	-348.900
CTG	-14.1505	-54.2200	-372.700
CTT	-16.7970	-55.9855	-353.500
CTA	-15.1555	-54.7480	-356.800
CAC	-14.2845	-54.1375	- -356.300
CAG	-14.1505	-54.2200	-372.700
CAT	-14.5525	-54.7975	-355.700
CAA	-14.6530	-54.9790	-351.500
GCC	-11.0350	-54.3355	-341.800
GCG	-10.7670	-52.9000	-345.600
GCT	-13.1455	-54.1540	-334.500
GCA	-12.4085	-53.9230	- 346.800
GGC	-11.0350	-54.3355	-341.800
GGG	-10.7000	- 56.1505	329.400
GGT	-11.1690	-55.3585	-347.900
GGA	-11.2025	-55.6390	-338.400
GTC	-13.7485	-54.5335	-340.700
GTG	-14.2845	-54.1375	-356.300
GTT	-15.9595	-54.3850	-347.300
GTA	-14.7200	-54.3685	-347.200
GAC	-13.7485	-54.5335	-340.700
GAG	-14.2845	-55.7875	-348.900
GAT	-14.8875	-54.7150	-341.100
GAA	-13.4135	-55.3750	-347.000
TCC	-11.2025	-55.6390	-338.400
TCG	-12.6765	-54.3850	-351.300
TCT	-13.5475	-55.7710	-342.000
TCA	-13.4470	-54.9295	-349.000
TGC	-12.4085	-53.9230	-346.800
TGG	-13.9830	-55.8865	-348.000
TGT	-13.6145	-54.6655	-351.600
TGA	-13.4470	-54.9295	-349.000
TTC	-13.4135	-55.3750	-347.000
TTG	-14.6530	-54.9790	-351.500
TTT	-17.1655	-55.4740	-347.800
TTA	-16.8975	-55.6225	-348.800
TAC	-14.7200	-54.3685	-347.200
TAG	-15.1555	-54.7480	-356.800
TAT	-17.1990	-55.2265	-355.800
TAA	-16.8975	-55.6225	-348.800
ACC	-11.1690	-55.3585	-347.900
ACG	-13.0450	-53.3785	-349.700
ACT	-14.0165	-53.7580	-340.400
AGC	-13.1455	-54.1540	-334.500
AGG	-13.9495	-56.2000	-344.700

**AGT**	-14.0165	-53.7580	-340.400
**ACA**	-13.6145	-54.6655	-1351.600
**AGA**	-13.5475	-55.7710	-342.000
**ATC**	-14.8875	-54.7150	-341.1000
**ATG**	-14.5525	-54.7975	-355.7000
**ATT**	-17.4000	-55.0285	-348.0000
**ATA**	-17.1990	-55.2265	-355.8000
**AAC**	-15.9595	-54.3850	-347.300
**AAG**	-16.7970	-55.9855	-353.500
**AAT**	-17.4000	-55.0285	-348.000
**AAA**	-17.1655	-55.4740	-347.800

Supplementary Method 1:
Explanation of the methodology used to calculate the physicochemical property features of dinucleotides and trinucleotides.

For every given sequences of lncRNA/mRNA, compute the transition matrix for dinucleotide and trinucleotide separately. Then divide each element of the row of the matrix by summation of the entire element of this particular row. Similarly, compute for each row for the matrix. Now This Revised matrix is used as a weight matrix. Using this weight matrix, we calculate the weighted mean physicochemical energy of lncRNA and mRNA for dinucleotides and trinucleotides.

Let a toy sequence of lncRNA/mRNA 5'GCTGTCGTT 3',
Divide this sequence into groups of trinucleotides as GCT GTC, GTT
and, group of dinucleotides as GC, TG, TC, GT
start calculating this sequence, by beginning trinucleotide as the first trinucleotide GCT, then the second trinucleotide GCT, and so on.

There are 64 possible trinucleotides and 16 possible dinucleotides that are equally probable because anyone from 64 or 16 can come in the start. So, the transition of trinucleotide in toy sequence looks like as:

Start-GCT-GTC-GTT
$S_k=(1/64)GCT^{physico}+(3/N \sum_{ij} V_j)$, and for Dinucleotides
Start-AC-TG-TC-GT
$S_k=(1/16)GCT^{physico}+(2/N \sum_{ij} V_j)$

Where V_j is the value of experimental physicochemical energy of dinucleotide or trinucleotide taken from the published paper mentioned above. Wij is the weight (weight of I $_{th}$ row and j $_{th}$ column) of weight (revise transition) matrix.

S_k is calculated weighted mean physicochemical energy of k_{th} mRNA or lncRNA sequence. $GCT^{physico}$ and $GC^{physico}$ are the experimental Physicochemical energy of the beginning trinucleotide or dinucleotide respectively, And the N length of the sequence.
Using this method calculate the weighted mean physicochemical energy of every sequence of lncRNA and mRNA gene.

Supplementary Method 2:
Base periodicity of lncRNA and mRNA
We have analyzed the peak of the power spectrum and SNR of peak spectrum. In bioinformatics, base periodicity has been explored to unravel biological features. Fourier spectrum of lncRNA and mRNA is defined using binary indicator function. U_a, where a is a symbol of nucleotide. $U_a(x_j)$= 1 if x_j= 1, otherwise 0, where x_j is the jth position of nucleotides in sequences of lncRNA/mRNA. Now, total spectrum is summation power spectrum of individual indicator function is given as:

$$S(f) = \sum_\alpha \frac{1}{N^2} \left(\sum_{j=1}^{N} U\alpha(xj)\exp(2\pi i f_j)\right)^2 \qquad (1)$$

Where, N is length of RNA sequence, f=k/N,k=1,2,3....N/2, and $_a s_a(f\ )$ is Σ a partial power spectrum of nucleotide. Also, signal-to-noise (SNR) of the peak at frequency 1/3 is

$$SNR = \frac{S(\frac{1}{3})}{avg(power} \qquad (2)$$

$$where\ , avg(power) = \frac{2}{N}(3)$$

Supplementary Table 3: Neural network setup for combination of features

Features Combination	Dimension of features	Hidden layers in neural network	Neurons in hidden layers
**DP**	3	2	4+2
**TP**	3	2	4+2
**DC**	15	4	16+8+4+2
**TC**	15	4	16+8+4+2
**BP**	2	2	4+2
**DPTP**	6 (3+3)	3	8+4+2
**BPDP**	5(2+3)	3	8+4+2

**BPTC**	15(2+13)	4	16+8+4+2
**BPDPTP**	8(2+3+3)	3	8+4+2
**BPDCTC**	15(2+7+6)	4	16+8+4+2
**BPDP**	5(2+3	3	8+4+2
**BPTP**	5(2+3)	3	8+4+2
**DPDC**	15(3+15	4	16+8+4+2
**DCTC**	15(8+7)	4	16+8+4+2
**TPTC**	15(3+12)	4	16+8+4+2
**TPTCDPDC**	15(3+5+3+4)	4	16+8+4+2

Supplementary explanation 1:

Neural network setup explanation and illustration:

We considered fifteen features for individual models. For illustration consider the type of feature combination is TPTCDPDC, with 8 symbols means it is composed of four features, containing TP, TC, DP, and DC. So, the total dimension of features is that 3 features of TP +5 features (first 5 PC of trinucleotides) + 3 features of DP and first 4 PC of dinucleotides. Neural network setup was implemented through software tool and techniques like keras. The first column of this table is a combination of features. The Second column of the Table contains the total number of hidden layers. These are the number of hidden layers between the input and output layers. The third column of this Table contains the number of artificial neurons in the hidden layers. For e.g. the neural network for TPTCDPDC contains 4 hidden layers with 16, 8, 4 and 2 neurons. The performance metrics for the above-constructed models are provided in the result section.

Supplementary Figure 1: flow chart of data preparation.

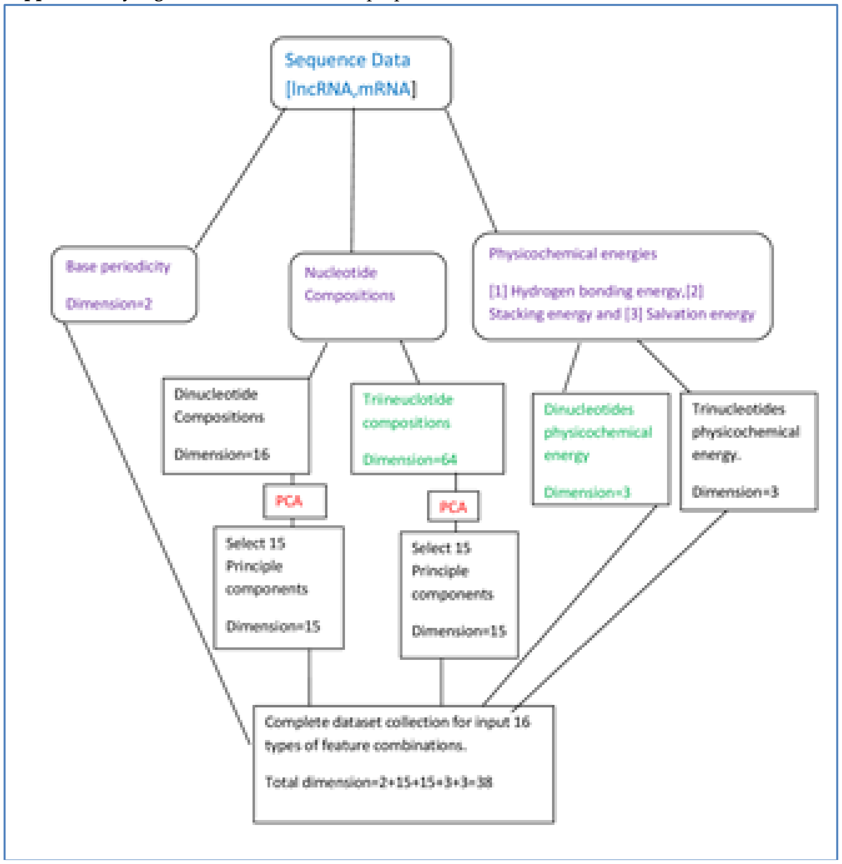

Supplementary explanation 2:

Detail of lognormal distribution fitting

MLE values of the lognormal distribution are listed in the following table 4. Although the distribution of peak spectrum at 1/3 of frequency and SNR for both RNA sequences have log-normal but slightly differ in parameters due to differences in inherent variability and diversity.

It is shown in Table 4 MLE value of parameters of μ are almost similar for a maximum peak in lncRNA as well as mRNA. There are a large number of sequences whose power spectrum values are a very small number, and natural logarithm is a negative value The natural logarithm of a number less than 1 is negative, This is a common characteristic of log-normal distributions when the data includes values close to zero. The similarity in μ values between the two sequence types suggests that they may share common statistical properties, at least in terms of their central tendency. The relatively larger values for SNR may be influencing the shape and location of the lognormal distributions. Higher SNR values may lead to shifts in the location μ or the scale sigma parameters of the lognormal distribution, affecting the estimated values of μ. Hence SNR of the sequence has more distinguishability than the maximum peak.

C. elegans have about 28532 mRNA sequence in the transcript file of NCBI and about 3152 lncRNA sequences in the NONECODE database. Few mRNAs have a lower SNR than 4, whenever more than 3000 lncRNA sequences have an SNR less than 4.So SNR can be confidence having a good distinguishable feature.

Supplementary Table 4: MLE values of parameters of log-normal distribution of SNR andpeak spectrum for lncRNA and mRNA sequences

Model organism	lncRNA-peak(μ/ σ)	mRNA-peak(μ/ σ)	lncRNA-SNR(μ/ σ)	mRNA-SNR(μ/ σ)
**Chimpanzee**	-5.648/0.808	-5.682/0.774	1.190/0.345	**2.012/0.714**
**Playtypus**	-5.310/0.668	-5.682/0.774	1.149/0.264	**2.0128/0.714**
**Zebrafish**	-5.595/0.674	-5.937/0.614	1.182/0.271	**1.879/0.679**
C.elegans	-5.189/0.735	-5.513/0.617	1.1687/0.3594	**1.884/0.769**
**Chicken**	-5.526/0.740	-6.184/0.894	1.202/0.310	**1.939/0.728**
**Cow**	-5.488/0.617	-5.770/0.729	1.088/0.189	**2.106/0.782**
Arabodopsis thaliana	-5.748 /0.721	-5.833/0.518	1.359/ 0.425	**1.792/0.604**
**Mix**	**-7.099/1.162**	**-6.512/1.019**	**-0.098/0.733**	**1.392/1.010**

Supplementary Table 5: Loss of original variance of dinucleotide/trinucleotide in principal components (pc)

Organism	RNA	15pc	12pc	7pc	6pc	5pc	4pc
Arabidopsis thaliana							
	lncRNA	0.191/0.713	0.279/0.870	0.571/ 1.44	0.601/1.554	1.44/1.645	**1.5484/2.043**
	mRNA	0.240/0.710	0.317/0.835	0.564/1.502	0.705/1.792	1.1840/1.814	**1.459/ 2.341**
C. elegans							
	lncRNA	0.254/0.801	0.393/0.997	1.471/0.828	0.928/1.568	1.064/1.785	**1.213 /2.046**
	mRNA	0.085/0.509	0.157/.625	0.324/1.299	0.383/1.511	0.480/2.175	**0.823 /2.836**
**Chicken**							
	lncRNA	0.216/0.452	0.279/0.520	0.499/0.790	0.551/0.831	1.017/1.149	**1.398/1.572**
	mRNA	0.114/0.433	0.139/0.583	0.293/1.444	0.350/1.546	0.699/2.0938	**1.105 /3.688**
**Chimpanzee**							
	lncRNA	0.194/0.438	0.241/0.543	0.369/ 0.789	0.789/1.141	1.221/1.350	**1.507/1.764**
	mRNA	0.0473/0.306	0.725/0.406	0.144/0.959	0.170/1.223	0.263/1.771	**0.6399/2.581**
**Cow**							
	lncRNA	0.291/0.524	0.329/0.596	0.485/ 0.915	0.493/0.954	1.132/ 1.238	**1.582/1.686**
	mRNA	0.155/0.461	0.218/0.580	0.375/1.545	0.530/1.695	0.614/2.060	**1.343/5.333**
**Platypus**							
	lncRNA	0.339/0.549	0.396/0.580	0.566/0.755	0.688/0.876	1.054/1.157	**1.632/ 1.753**
	mnrRNA	0.066/4.331	0.120/0.507	0.3695/1.148	0.476/1.373	0.736/1.518	**0.734/3.443**
**Zebrafish**							
	lncRNA	0.324/0.752	0.462/0.819	0.805/1.140	0.462/1.191	1.291/1.355	**1.434/1.492**
	**mRNA**	**0.222/0.609**	**0.305/0.715**	**0.505/1.361**	**0.585/1.473**	**0.844/2.008**	**1.187/5.590**

pplementary Table 6: The metrics of neural networks for the classification of various combination of features of eukaryotic organisms Chimpanzee and *C. elegans* using SMOTE

Organism	Chimpanzee					
**Combination of feature types**	**Accuracy**	**Precision**	**recall**	**ROC**	**PRC**	**F1score**
**DP**	0.7874	0.7862	0.7856	0.86261	0.8712	**0.7855**
**TP**	0.7722	0.7752	0.76490	0.81682	0.8201	**0.7701**
**DPTP**	0.8296	0.8315	.82392	0.90242	0.9044	**0.8264**
**DC**	0.9500	0.9511	0.9490	0.9844	0.9853	**0.9496**
**TC**	0.9584	0.96900	0.9464	0.98918	0.9901	**0.95729**
**DCTC**	0.9916	0.9915	0.991515	0.9986	0.9978	**0.99165**
**BP**	0.8326	0.8015	0.8875	0.9060	0.9060	**0.8423**
**BPDP**	0.8754	0.8484	0.9137	0.9393	0.927	**0.8799**
**BPTP**	0.8465	:0.8264	0.8820	0.91839	0.9016	**0.8533**
**BPDPTP**	0.8760	0.8641	0.8991	0.9450	0.9386	**0.8812**
**BPDC**	0.9990	0.9991	0.9988	0.9997	0.9998	**0.9990**
**BPTC**	0.9987	0.9991	0.9983	0.9998	0.9999	**0.9987**
**BPDCTC**	0.9983	0.9988	0.9977	0.9998	0.9997	**0.9983**
**DPDC**	0.97986	0.98139	0.97841	0.99719	0.9963	**0.9800**
**TPTC**	0.99722	0.9969	0.99750	0.9995	0.9995	**0.9972**
**DPTPDCTC**	0.9984	0.9972	0.9997	0.9992	0.9985	**0.9984**
Organism	***C.elegans***					
**Combination of feature Types**	Accuracy	Precision	Recall	ROC	PRC	**F1score**
**DP**	0.7788	0.8004	0.7444	0.8595	0.8448	**0.7714**
**TP**	0.7915	0.7602	0.8513	0.8763	0.8680	**0.8027**
**DPTP**	0.8020.	0.7996	0.7955	0.8885	0.8887	**0.7958**
**DC**	0.8807	0.8712	0.8943	0.9556	0.9567	**0.8823**
**TC**	0.9176	0.9112	0.9264	0.9731	0.9708	**0.9184**
**DCTC**	0.9888	0.9887	0.9892	0.9988	0.9968	**0.9889**
**BP**	0.8030	0.7884	0.8321	0.8916	0.8793	**0.8097**
**BPDP**	0.8106	0.8214	0.7881	0.8992	0.8959	**0.8044**
**BPTP**	0.8253	0.821471	0.8314	0.9079	0.9011	**0.8314**
**BPDPTP**	0.8345	0.8247	0.8507	0.9245	0.9267	**0.8375**
**BPDC**	0.9978	0.9989	0.9968	0.9997	0.99970	**0.9978**
**BPTC**	0.9986	0.9982	0.9991	0.9999	0.99980	**0.9986**
**BPDCTC**	0.9993	0.99982	0.99894	0.99964	0.99978	**0.9993**
**DPDC**	0.9940	0.9930	0.9947	0.9993	0.9989	**0.9938**

**TPTC**	0.9991	0.9982	1.000	0.9997	0.9994	**0.9991**
**DPTPDCTC**	**0.9999**	**0.9999**	**0.9993**	**0.9997**	**0.9999**	**0.9995**

Supplementary Table 7: The metrics of neural networks for the classification of various combination of features of eukaryotic organisms Cow and Platypus using SMOTE.

Organism	Cow					
**Combination of feature Type**	Accuracy	Precision	Recall	ROC	PRC	**F1score**
**DP**	0.8365	0.8552	0.8095	0.9034	0.8962	**0.83150**
**TP**	0.8815	0.8917	0.8669	0.9464	0.9396	**0.87889**
**DPTP**	0.9013	0.91481	0.8834	0.9617	0.9660	**0.8988**
**DC**	0.8421	0.8091	0.8904	0.9229	0.9144	**0.84781**
**TC**	0.9673	0.9633	0.9721	0.9947	0.9935	**0.9677**
**DCTC87**	0.99138	0.9885	0.9942	0.9982	0.99726	**0.9915**
**BP**	0.9157	0.8958	0.9392	0.9734	0.9707	**0.91701**
**BPDP**	0.92547	0.9168	0.93612	0.9799	0.97843	**0.9263**
**BPTP**	0.93833	0.9441	0.9316	0.9850	0.9837	**0.93783**
**BPDPTP**	0.94131	0.93119	0.9539	0.98632	0.9858	**0.94245**
**BPDC**	0.9987	0.9976	0.9997	0.9999	0.999994	**0.99874**
**BPTC**	0.99946	0.99934	0.99956	0.999997	0.9999973	**0.99945**
**BPDCTC**	0.99968	0.99935	1.0	0.99989	0.999777	**0.99967**
**DPDC**	0.9885	0.98271	0.99473	0.9980	0.9965	**0.9885**
**TPTC**	0.9990	0.9985	0.9995	0.9996	0.99951	**0.9990**
**DPTPDCTC**	0.9986	0.9978	0.9993	0.9998	0.9997	**0.9986**
Organism **DP**	**Platyps** 0.87801	0.89061	0.8579	0.9434	0.94967	**0.8730**
**TP**	0.8612	0.8950	0.8193	0.8949	0.9284	**0.8552**
**DPTP**	0.9288	0.9301	0.9272	0.9820	0.9839	**0.9291**
**DC**	0.9591	0.9704	0.9704	0.9922	0.9934	**0.9591**
**TC**	0.9830	0.9875	0.9788	0.9976152	0.99789	**0.9831**
**DCTC**	0.9946	0.9964	0.9929	0.9984	0.9987	**0.99447**
**BP**	0.96275	0.93993	0.98887	0.99049	0.98989	**0.963782**
**BPDP**	0.966324	0.95116	0.98302	0.99129	0.99015	**0.96683**
**BPTP**	0.9694	0.963963	0.974055	0.99481	0.99494	**0.96898**
**BPDPTP**	0.97033	0.96236	0.97907	0.99315	0.98955	**0.970646**
**BPDC**	0.99776	0.99954	0.99594	0.99997	0.999979	**0.96683**
**BPTC**	0.99866	1	0.99726	0.9999977	0.99999773	**0.99933051**
**BPDCTC**	0.999553	0.99954	0.999547	0.99976	0.9998527	**0.9995478**
**DPDC**	0.9977	0.9990	0.9963	0.9992	0.9995	**0.9977**
**TPTC**	0.9968	0.9942	0.99955	0.9971	0.9941	**0.9968**
**DPTPTC**	**0.9975**	**0.9981**	**0.9967**	**0.9995**	**0.9996**	**0.9974**

Supplementary Table 8: The metrics of neural networks for the classification of various combinations of features of eukaryotic organisms Zebra fish and *Arabidopsis thaliana* using SMOTE.

Organism	Zebrafish					
**Combination of feature Type**	Accuracy	Precision	Recall	ROC	PRC	**F1score**
**DP**	0.81240	0.8006	0.83214	0.87954	0.8600	**0.8159**
**TP**	0.87347	0.85960	0.89232	0.943174	0.9310	**0.8752**
**DPTP**	0.83904	0.87527	0.79100	0.9152	0.91825	**0.83112**
**DC**	0.7169	0.69269	0.7705	0.80038	0.777607	**0.7292**
**TC**	0.87558	0.8632	0.89239	0.947443	0.941089	**0.8763**
**BP**	0.86519	0.82765	0.91633	0.94021	0.92167	**0.869742**
**BPDP**	0.86356	0.84899	0.8817	0.940132	0.93377	**0.865081**
**BPTP**	0.86681	0.847151	0.88818	0.941859	0.9355	**0.94185**
**BPDPTP**	0.8629202	0.831203	0.910185	0.94429	0.93964	**0.868903**
**BPDC**	0.9983	0.99677	1.0	0.99966	0.9993	**0.998383**
**BPTC**	0.99902	0.99934	0.998691	0.999997	0.999997	**0.9990186**
**BPDCTC**	0.99918	0.998398	1.0	0.99966	0.999350	**0.999198**
**DCTC**	0.98554	0.98094	0.990218	0.9979	0.9968	**0.98555**
**DPCC**	0.98424	0.9758	0.9932	0.9965	0.9948	**0.9847**
**TPTC**	0.9988	0.9983	0.9993	0.999819	0.99965	**0.9988**
**DPTPTC**	0.9988	0.99803	0.99967	0.9995	0.9989	**0.99885**
organism **DP**	***Arabidopsisthaliana*** 0.6925	0.7232	0.6172	0.7464	0.7242	**0.6637**
**TP**	0.6830	0.7179	0.5998	0.7254	0.7271	**0.6519**
**DPTP**	0.7136	0.7086	0.7282	0.7810	0.7747	**0.7161**
**DC**	0.8242	0.8113	0.8441	0.9071	0.8958	**0.8266**
**TC**	0.9229	0.9052	0.9420	0.9762	0.9719	**0.9232**
**DCTC**	0.9984	0.9981	0.9988	0.9999	0.9998	**0.9984**
**BP**	0.7170	0.7029	0.7545	0.7870	0.7638	**0.7278**
**BPDP**	0.7807	0.7741	0.7991	0.8565	0.8302	**0.7864**
**BPTP**	0.8119	0.7787	0.8649	0.8886	0.8546	**0.8196**
**BPDPTP**	0.8244	0.7977	0.8697	0.8935	0.85438	**0.8322**
**BPDC**	0.9810	0.9702	0.9924	0.9951	0.9930	**0.9812**
**BPTC**	0.9757	0.9638	0.9887	0.9956	0.9939	**0.9761**
**BPDCTC**	0.9975	0.9966	0.9985	0.9998	0.9997	**0.9975**

**DPDC**	0.9812	0.9802	0.9835	0.9970	0.9977	**0.9832**
**TPTC**	0.9919	0.9872	0.9967	0.9991	0.9985	**0.9919**
**DPTPDCTC**	**0.9989**	**0.9981**	**0.9997**	**0.9990**	**0.9996**	**0.9989**

Supplementary Table 9: The metrics of neural networks for the classification of various combination features of eukaryotic organisms Chicken and mix sequences of species *C. elegans*, Chicken and platypus using with SMOTE/without SMOTE.

Organism	Chicken					
F**eatures**	Accuracy	Precision	Recall	ROC	PRC	**F1score**
**DP**	0.8463/0.83711	0.8570/0.8320	0.8229/0.8360	0.9264/0.91024	0.9275/0.90957	**0.8395/0.83399**
**TP**	0.8464/0.83174	0.8694/0.8477	0.8180/0.8159	0.9289/0.91227	0.9362/0.91743	**0.8411/0.83149**
**DPTP**	0.8634/0.8496	0.8805/0.8446	0.8383/0.85081	0.9376/0.9222	0.9425/0.9211	**0.8570/0.8473**
**DC**	0.8332/0.8290	0.8333/0.8158	0.8250/0.8485	0.9126/0.90757	0.90540/0.90757	**0.8280/0.831828**
**TC**	0.9157/0.8845	0.9096/0.8769	0.92473/0.8932	0.971830.9530/	0.9678/0.9505	**0.91773/0.88497**
**DCTC**	0.97801/0.97941	0.9768/0.9776	0.97953/0.98273	0.9966/0.9960	0.9950/0.9946	**0.9782/0.9801**
**BP**	0.8875/0.8705	0.8823/0.8489	0.8914/0.9041	0.9568/0.94012	0.9541/0.94012	**0.8868/0.875630**
**BPDP**	0.8984/0.8887	0.8810/0.8754	0.9155/0.90903	0.9619/0.9502	0.9590/0.94079/	**0.8979/0.891898**
**BPTP**	0.8824/0.87022	0.8626/0.8724	0.9131/0.875	0.9562/0.9445	0.9543/0.9437	**0.8871/0.873698**
**BPDPTP**	0.8972/0.8911	0.8764/0.8710	0.9243/0.9165	0.9601/0.9531	0.9582/0.9477	**0.899712/0.8931**
**BPDC**	0.9974/0.99701	0.9988/0.9953	0.9961/0.9988	0.9997/0.9999	0.9995/0.999982	**0.9974/0.99704**
**BPTC**	0.9972/0.99373	0.9968/0.9945	0.9976/0.99275	0.9995/0.99925	0.9997/0.999497	**0.99726/0.99362**
**BPDCTC**	0.9974/0.9985	0.9988/0.9993	0.9961/0.99758	0.9997/0.9996	0.9998/0.9998	**0.9974/0.9984**
**DPDC**	0.9896/0.9895	0.9918/0.98983	0.9876/0.9892	0.99879/0.9978	0.9985/0.9971	**0.9897/0.98951**
**TPTC**	0.9980/0.9976	0.9972/0.9952	0.9988/1.0	0.99957/0.9999879	0.9991/0.99998	**0.9980/0.99759**
**DPTPDC TC**	0.9990/0.99970	1.0/0.99938	0.9980/1.0	1.0/0.9999	1.0/0.9999	**0.99902/0.99968**
Organism<br>**DP**	**mixtype**<br>0.789611/0.79154	0.78612/0.8286	0.79373/0.73159	0.861178/0.86976	0.86015/0.87754	**0.7898/0.7770**
**TP**	0.7859/0.76260	0.80369/0.7756	0.75629/0.7382	0.84603/0.81815	0.8392/0.7894	**0.7792/0.75643**
**DPTP**	0.8103/0.81717	0.81931/0.85030	0.79425/0.76540	0.88382/0.89422	0.89166/0.9055	**0.8065/0.80561**
**DC**	0.8381/0.90904	0.8389/0.9171	0.8365/0.89783	0.9206/0.96753	0.91992/0.9699	**0.8376/0.90736**
**TC**	0.91656/0.9978	0.9134/0.9979	0.92274/0.9977	0.97415/0.99941	0.97414/0.99950	**0.9180/0.9584**
**DCTC**	0.99037/0.99972	0.98920/1.0	0.99154/0.99944	0.998984/0.9999	0.9986/0.999999	**0.99036/0.9997**
**BP**	0.8341/0.79981	0.8061/0.7435	0.8808/0.90989	0.90765/0.87198	0.887455/0.83723	**0.8924/0.8183**
**BPDP**	0.8590/0.86375	0.83720/0.8428	0.89071/0.8911	0.9318/0.9404	0.9231/0.93637	**0.8631/0.8662**
**BPTP**	0.87095/0.84051	0.85326/0.81961	0.89708/0.87428	0.9398/0.92085	0.9317/0.913705	**0.87458/0.8855**
**BPDPTP**	0.87113/0.87147	0.85104/0.85293	0.8982/0.89952	0.9403/0.9477	0.9316/0.94775	**0.8739/0.8756**
**BPDC**	0.9936/0.9986	0.9920/0.9988	0.99541/0.9983	0.9993/0.99989	0.9990/0.9998	**0.9937/0.9985**
**BPTC**	0.99248/0.9992	0.9885/0.9998	0.99641/0.99873	0.9989/0.99981	0.99841/0.9998	**0.9924/0.9992**
**BPDCTC**	0.99750/0.99926	0.99681/1.0	0.99818/0.9985	0.9997/0.9996	0.99954/0.9998	**0.9974/0.9989**
**DPDC**	0.98847/0.98879	0.98571/0.9864	0.99135/0.9911/	0.9989/0.9990	0.99851/0.99867	**0.98852/0.9887**
**TPTC**	0.99853/0.99972	0.9981/0.99981	0.99892/0.99963	0.99987/0.999906	0.9997/0.99994	**0.99849/0.9997**
**DPTPDC TC**	**0.9990/0.9993**	**0.9983/0.99927**	**0.99965/0.99945**	**0.99971/0.999990**	**0.99945/0.99999**	**0.9989/0.99935**

Supplementary Figure 2 : Classification metrics for mixed sequences of seven considered organism using SMOTE (second approach)

a)

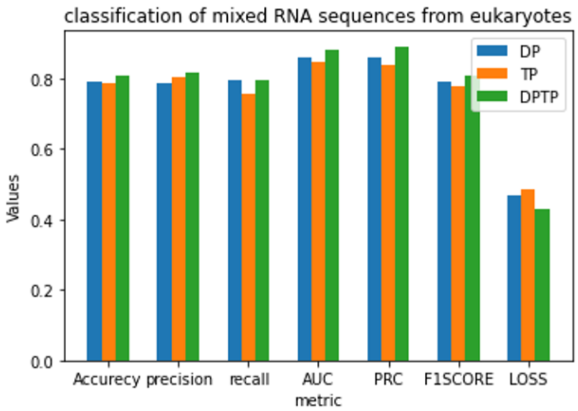

b)

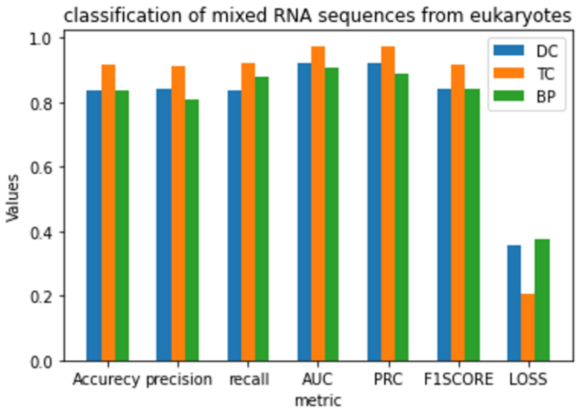

c)

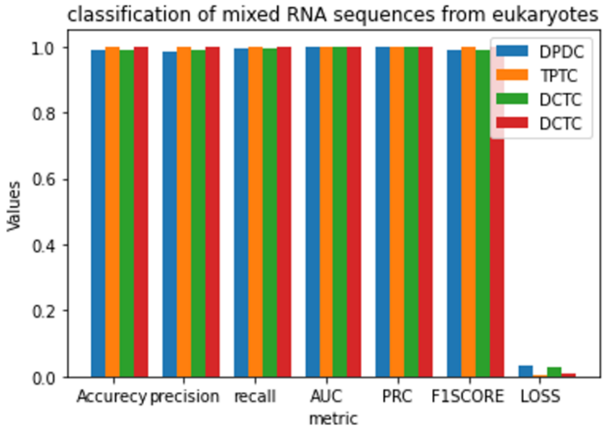

d)

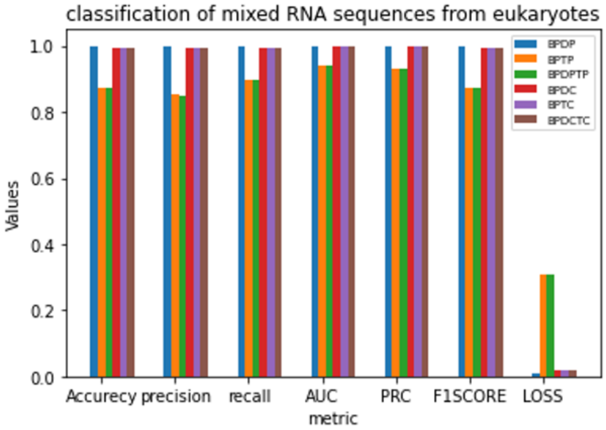

Figure 2.a : Feature based on physicochemical energy, **Figure 2.b** : feature based on PCA-based composition and base periodicity , **Figure2.c** : Combined feature based on physico -chemical energy and PCA-based compositions. **Figure 2.d** : Combined feature based on physicochemical energy, base periodicity and PCA-based composition.
